# Cutting a Long Intron Short: Recursive Splicing and Its Implications

**DOI:** 10.3389/fphys.2016.00598

**Published:** 2016-11-29

**Authors:** Theodore Georgomanolis, Konstantinos Sofiadis, Argyris Papantonis

**Affiliations:** Chromatin Systems Biology Laboratory, Center for Molecular Medicine, University of CologneCologne, Germany

**Keywords:** recursive splicing, variant U1 RNAs, processing, exon definition, RNA polymerase, co-transcriptional

## Abstract

Over time eukaryotic genomes have evolved to host genes carrying multiple exons separated by increasingly larger intronic, mostly non-protein-coding, sequences. Initially, little attention was paid to these intronic sequences, as they were considered not to contain regulatory information. However, advances in molecular biology, sequencing, and computational tools uncovered that numerous segments within these genomic elements do contribute to the regulation of gene expression. Introns are differentially removed in a cell type-specific manner to produce a range of alternatively-spliced transcripts, and many span tens to hundreds of kilobases. Recent work in human and fruitfly tissues revealed that long introns are extensively processed cotranscriptionally and in a stepwise manner, before their two flanking exons are spliced together. This process, called “recursive splicing,” often involves non-canonical splicing elements positioned deep within introns, and different mechanisms for its deployment have been proposed. Still, the very existence and widespread nature of recursive splicing offers a new regulatory layer in the transcript maturation pathway, which may also have implications in human disease.

## Introduction

The interruption of a gene's open reading frame by a non-protein-coding sequence, i.e., by an intron, is an exclusive feature of eukaryotes. It is now thought that the course of evolution has brought about such an exon-intron gene structure concomitantly with the emergence and diversification of multicellular eukaryotes (Rogozin et al., 2012) and the need for complex gene regulation (Jeffares et al., 2008). However, introns are not “genomic junk”; they have been shown to confer important regulatory capacity, they typically carry *cis*-regulatory elements important for both transcription and splicing (Wang and Burge, 2008; Levine, 2010), and have even been found to be partially or fully coding (Marquez et al., 2015).

An average mammalian gene will contain 8–9 introns; >3000 human introns are longer than 50 kbp, and >1200 longer than 100 kbp (Bradnam and Korf, 2008; Shepard et al., 2009). This poses the following problem. In long introns the three sites reactive in a splicing reaction (i.e., the 5′ splicing site, the branch-point, and the 3′ splice site; Hollander et al., 2016) will be separated by large stretches of RNA sequence. Thus, it becomes difficult to explain how the sites required for splicing can find one another in three-dimensional space, or how a primary transcript spanning tens to hundreds of kbp can be protected from unspecific hydrolytic cleavage in the time it takes an RNA polymerase to copy it as one continuous RNA (e.g., at an average speed of 3 kbp/min, >30 min are required to fully transcribe a 100 kbp-long intron; Wada et al., 2009).

An elegant solution to this problem was proposed for Drosophila long introns—recursive splicing (RS). According to this, long introns are removed in a stepwise manner by splicing at intronic sites that carry the expected acceptor and donor splice sequences in the three gene examples studied (consensus sequence: 5′-(Y)_n_NCAG|GTAAGT-3′; the vertical line represents the splicing junction; Burnette et al., 2005). Similarly, a “zero-length” exon was identified between the 2nd and 3rd exon of the rat α-tropomyosin gene (Grellscheid and Smith, 2006), as well as “dual specificity” splicing sites in human pre-mRNAs (Zhang et al., 2007). Still, despite computational efforts (Shepard et al., 2009), the RS concept was not verified in humans until 2015. A study in human primary endothelial cells (Kelly et al., 2015), followed by two back-to-back studies across Drosophila tissues (Duff et al., 2015) and in human brain (Sibley et al., 2015), revealed that RS is a conserved and widespread splicing mechanism. Nonetheless, the fruitfly and human RS-sites differ in composition, and their molecular recognition and processing remains unknown. Here, we discuss different scenarios by which recursive splicing might manifest, as well as its potential implications in gene expression regulation and deregulation.

## Models for the processing of recursive splicing intermediates

The idea that intronic sequences are not evolutionarily constrained, because they do not code for proteins, pervades our thinking; however, the conservation of parts of these non-coding sequences between three diverse mammalian genomes (human, whale, and seal) amounts to almost 50% in pairwise comparisons, and to 28% amongst the three taxa (Hare and Palumbi, 2003). This hints to the existence of underappreciated classes of intronic regulatory elements. Recent work on recursive splicing in human cells (Kelly et al., 2015; Sibley et al., 2015) in part confirms this by using deep RNA sequencing and data analysis to find potential “ratchet” RS points. A large number of RS-sites was discovered (albeit different in the two studies, due to the different approaches and cutoffs used), the conservation of which was higher than that of similar, adjacent, intronic regions. These do not carry the consensus sequence identified in Drosophila, but rather one that contains a typical acceptor site followed by a donor sequence that is not the expected GT/GC/GA in >60% of cases (Kelly et al., 2015). This, of course, raises the question of how these non-canonical sites are recognized by the splicing machinery and processed accurately to produce a mature messenger RNA (although RNase R-resistant lariats as a result of recursive splicing were detected; Duff et al., 2015; Kelly et al., 2015).

One scenario could be that the vast majority of RS events detected, especially those with non-GT sequences at donor sites, represent “dead-end” products targeted for degradation. But, in human primary endothelial cells, a number of evidence does not concur with this scenario. First, the ~2400 RS high-confidence events recorded occur at ~15% the level of primary transcription; second, targeted genome editing of three different RS-sites in the 134 kbp-long intron of the *SAMD4A* gene showed that they are necessary for efficient mRNA production; third, knocking-down exosome components did not affect the levels of RS intermediates, either GT- or non-GT-containing (Kelly et al., 2015). Thus, splicing at RS-sites occurs at significant levels, is widespread, and does not appear linked to exosomal degradation, but rather to RNA maturation.

**Figure 1:**
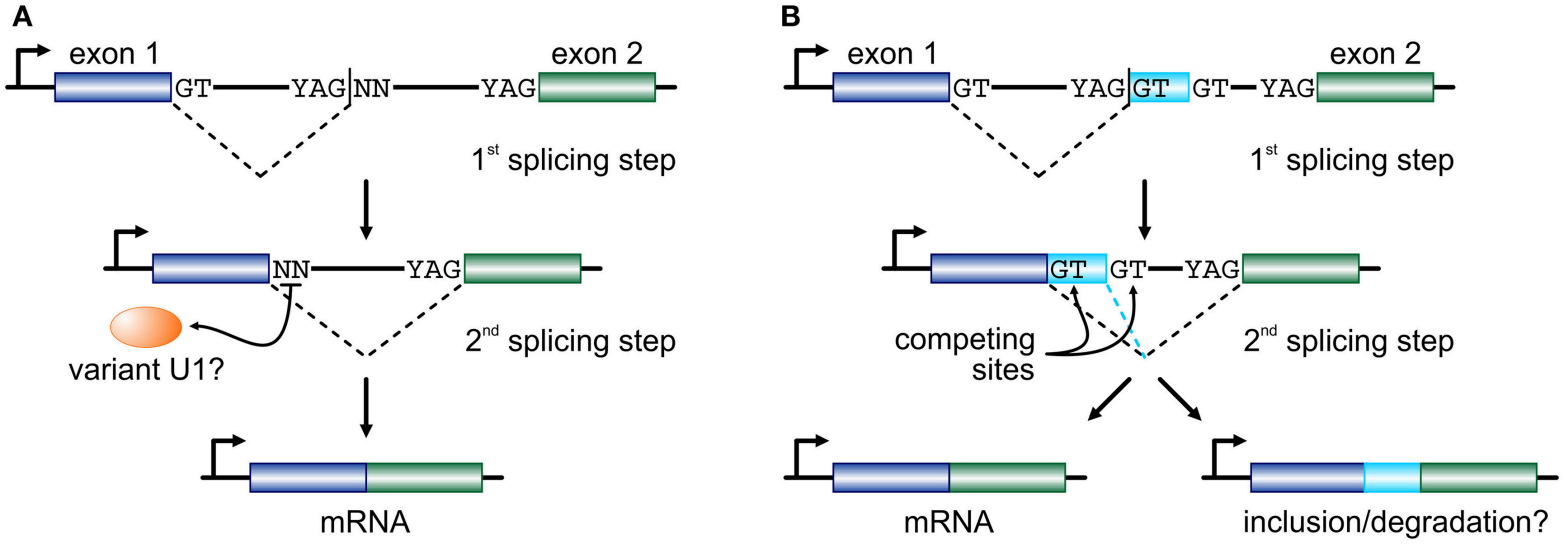
**Two models for recursive splicing processing. (A)** Two consecutive exons (blue and green boxes) are separated by a long intron which contains an RS-site with a canonical RS acceptor site and a non-canonical RS donor (YAG|NN). The GT at the 3′ end of exon 1 splices into the acceptor sequence of the RS-site, and the non-canonical NN sequence now acts as a splice donor in the 2nd splicing step to splice the two exons together. The recognition of this non-canonical splice site is presumably mediated by a variant U1 RNA (orange oval). **(B)** In a similar setup, where only RS-sites with a canonical GT donor dinucleotide are considered, the 1st splicing step occurs just as before. But, now exon 1 is spliced onto a putative cryptic or micro-exon (light blue box) that has another GT donor further downstream. Then, competition between the two donor sites determines whether the cryptic/micro-exon will be included in the mature RNA or not. The fate of the mRNA carrying this extra short sequence might involve degradation.

If RS intermediates lie on the productive pathway of mRNAs, the dinucleotide immediately downstream of an RS-junction will subsequently need to act as an efficient splicing donor. In endothelial cells, ~45% of RS-sites encode a GN dinucleotide and it has been shown that they can efficiently function as donors provided strong acceptor and “splicing enhancer” sequences also partake in that reaction (Twigg et al., 1998; Thanaraj and Clark, 2001; Dewey et al., 2006). For the remaining 55% of RS-sites, a combination of mechanisms might come into play. We now know that the U1-containing snRNPs, designed to identify the GT donor dinucleotide, are able to expand their base-pairing repertoire via mispairing (Roca et al., 2012; Tan et al., 2016). We have also come to find out that the human genome encodes a large number of “variant” U1 snRNAs (Kyriakopoulou et al., 2006; O'Reilly et al., 2013). Their expression is markedly higher in primary, embryonic, and pluripotent cells (O'Reilly et al., 2013; Kelly et al., 2015; Vazquez-Arango et al., 2016) and they are able to form proper RNPs *in vitro* (Somarelli et al., 2014). In endothelial cells, the repertoire of expressed variant U1, together with the minor spliceosome (Turunen et al., 2013), would suffice for the recognition of the vast majority of all non-canonical RS donor dinucleotides recorded (Kelly et al., 2015). In addition, efficient splicing has been shown to occur independently of U1-mediated recognition (Raponi and Baralle, 2008) or of the physical continuity of the nascent transcript (via “exon tethering”; Dye et al., 2006). With the aforementioned into account, we propose that long human introns are cotranscriptionally removed by splicing at RS-sites that may equally carry a canonical or a non-canonical donor dinucleotide, before the two flanking exons are joined together (Figure 1A).

Another model, proposed on the basis of data from human brain, sees RS-sites as a means for establishing a “binary splicing switch” (Sibley et al., 2015). However, it is worth noting here that this study focuses specifically on RS-sites that conform to the YAG|GT consensus, and thus investigated ~400 such junctions. According to this model, each RS-site may also act as an RS-exon whereby the GT dinucleotide immediately downstream of the splice site will compete with an alternative GT further downstream for splicing into the canonical acceptor site at the 3′ end of the long intron. This inter-site competition determines whether the very short RS-exon sequence will be retained as part of the final spliced transcript or not (Figure 1B; a mechanism similar to “intrasplicing”; Parra et al., 2008). It is suggested that inclusion of such RS-exons will target the mature transcript for degradation, as they encode premature termination codons (Sibley et al., 2015). However, their inclusion (if in-frame) will act on top of alternative splicing, and brain tissue was shown to be uniquely prone to the inclusion of microexons into mature mRNAs (Scheckel and Darnell, 2015), and this may not be perfectly reconciled with this RS model. Still, despite their differences, both models favor “noisy splicing,” which is thought to drive mRNA isoform diversity in human cells (Pickrell et al., 2010).

## Regulatory and disease implications of recursive splicing

**Figure 2:**
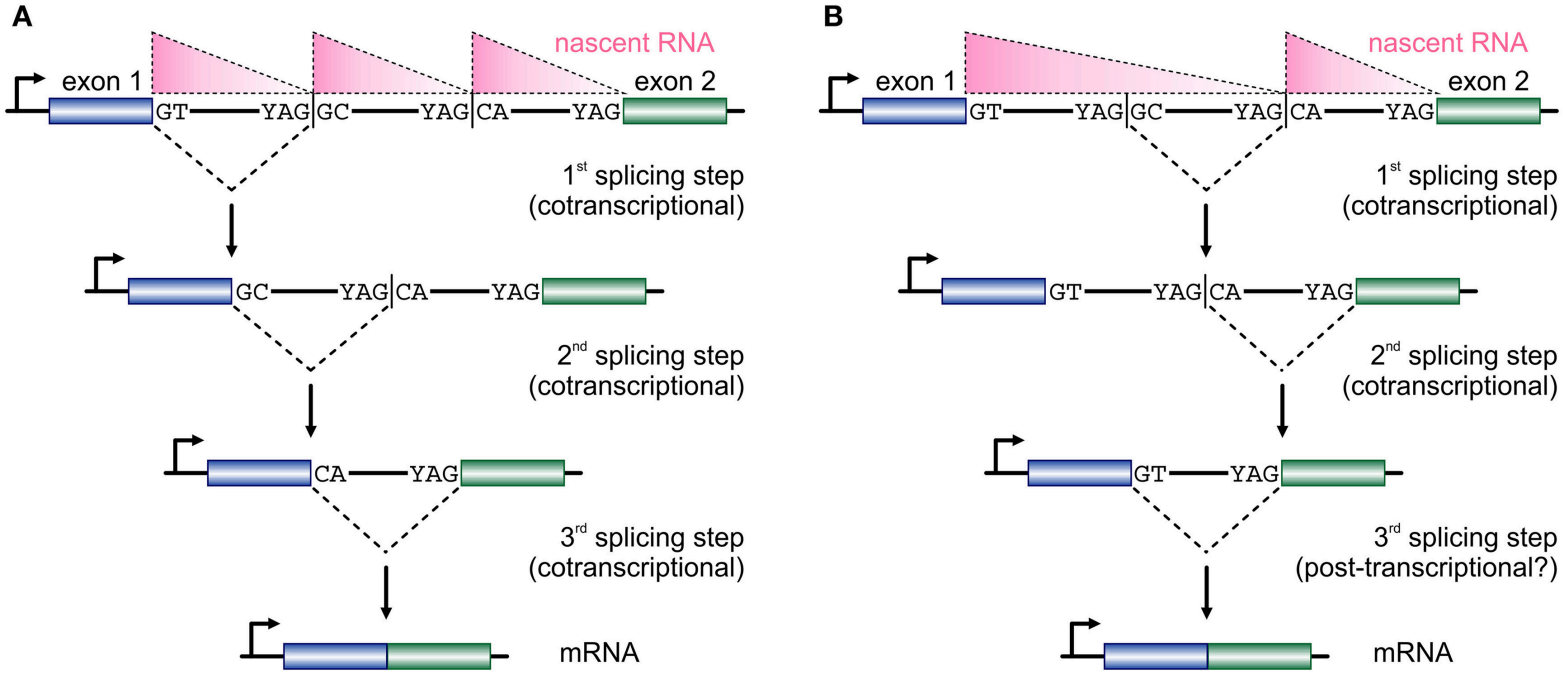
**Two models for temporal progression of recursive splicing. (A)** Two consecutive exons (blue and green boxes) are separated by a long intron which contains two RS-sites with canonical RS acceptor sites and non-canonical RS donors. Typically, nascent RNA profiles (pink triangles) along such long introns display a “saw-tooth” pattern. The GT at the 3′ end of exon 1 splices into the first RS-site, and the non-canonical GC sequence now acts as a splice donor in the 2nd splicing step into the next RS-site, before the two exons are spliced together after the RS-sites are utilized in an ordered, co-transcriptional, manner. **(B)** In a similar setting RS-sites are utilized in a non-ordered, nested, manner, which cannot be fully co-transcriptional and is also reflected on the distribution of nascent RNA. First, the intronic segment between the two RS-sites is removed, the splicing of the RS-donor into the acceptor at exon 2 occurs, before the two exons are spliced together.

The size of first introns in higher eukaryotes is such that, on average, exceeds all other downstream introns in length (Bradnam and Korf, 2008). This structural property of eukaryotic genomes has been linked with programmed delays in gene transcription cycles (Swinburne and Silver, 2008). As a result, the preferential positioning of RS-sites in such long introns (Kelly et al., 2015; Sibley et al., 2015) creates a novel regulatory layer for the processing of the nascent transcripts copied from these loci. Given that the majority of splicing in human cells occurs cotranscriptionally (Aitken et al., 2011; Tilgner et al., 2012), it would be reasonable to assume that the RS-junctions in one long intron are used successively at more or less the moment they are produced by the RNA polymerase (Figure 2A). This is supported by the study of TNF-inducible *SAMD4A*; upon induction, nascent RNA production progresses synchronously along its first intron and intronic RNA FISH fails to return evidence in favor of a single, long, transcript from this intron (Wada et al., 2009; Kelly et al., 2015). Intermediate splicing products at the 8 RS-sites in this 134-kbp intron appear and disappear in sync with the production of nascent RNA, and the half-life of each such RS-intermediate is ~1/15 the time it takes the RNA polymerase to fully transcribe this intron (Kelly et al., 2015). This evidence, plus the “saw-tooth” patterns observed in brain RNA-seq data (Sibley et al., 2015; see Figure 2), are in support of the successive use of RS-sites. Nonetheless, there have been reports of non-ordered (“nested”) use of such sites (Suzuki et al., 2013; Gazzoli et al., 2016), whereby the RS-sites can engage in splicing reactions decoupled from cotranscriptionality and in which long primary transcripts survive degradation (Figure 2B). In fact, such decoupling of RS has been proposed for yeast splicing (Lopez and Séraphin, 2000).

Another question that arises is: Are the RS-sites in a given long intron all used in every transcription cycle or is their usage more stochastic? Again, studies from the *SAMD4A* locus using CRISPR-Cas9 technology (Ran et al., 2013) to specifically mutate 3 RS-sites, showed that abolishing any one RS-site results in a 35–50% reduction in mRNA levels (Kelly et al., 2015). Similarly, reducing RS-site usage by antisense oligonucleotides in the zebrafish *cadm2a* gene led to a ~2-fold reduction in its mRNA levels *in vivo* (Sibley et al., 2015). These results (albeit based a limited number of example loci) point to a stochastic usage of multiple RS-sites along one intron and/or to compensatory mechanisms that prevent a complete loss of mRNA output. Additionally, it is necessary to investigate the connection between RS, exon skipping, and the formation of circular RNAs from a given gene locus, as they could all be functionally linked (Kelly et al., 2014).

RS-sites were found to be more conserved than equivalent intronic regions of similar composition in humans (Kelly et al., 2015; Sibley et al., 2015), and this hinted in favor of their functional role. As more than 90% of human genetic variation maps outside protein-coding regions, at inter- or intragenic sequences, and >40% maps within introns (Maurano et al., 2012), it is attractive to hypothesize that mutations at RS-sites may contribute to disease manifestation. Splicing defects are now well-established contributors in various diseases (Chabot and Shkreta, 2016), and RS, yet another layer of splicing regulation, remains unexplored. In fact, when we intersected a list of high-confidence RS-sites from human brain (Sibley et al., 2015) or endothelial cells (Kelly et al., 2015) to an ensemble of all putatively disease-causative human SNPs, they overlapped (within the 40 preceding the RS-junction) those associated with neurological (e.g., Parkinson's disease, cognitive performance) or circulatory disorders/traits (e.g., retinal vascular caliper, blood pressure), respectively, more than what was expected by chance (A. Papantonis; unpublished data). Such a potential role of RS should be further investigated in both disease models and in GWAS datasets, as it can—in conjunction with alternative splicing—impact heavily on the mRNA isoform that a given cell generates.

## Conclusions and outlook

We think that there is still much to be discovered about the molecular basis and the regulatory implications of recursive splicing. The presence of non-canonical splicing sequences at RS-sites, the possibility of splice-site competition, the proposed involvement of U1 variants, even the cotranscriptional and/or non-sequential processing of long introns all need to be systematically dissected. To cite just a few pertinent questions: How widespread is recursive splicing across mammalian tissues and developmental stages? Is it affected once cell homeostasis is challenged, and how does this affect transcript maturation? How are RS-sites defined, recognized, and marked epigenetically? Are they being utilized in a stochastic or a deterministic temporal order? Addressing these questions, amongst others, will be important for understanding this unforeseen regulatory layer of transcript processing in higher eukaryotes.

## Author contributions

TG, KS, and AP reviewed the bibliography and wrote the manuscript.

## Funding

This work is supported by the Deutsche Forschungsgemeinschaft via the SPP1935 Priority Program, and by CMMC intramural funding (both awarded to AP).

### Conflict of interest statement

The authors declare that the research was conducted in the absence of any commercial or financial relationships that could be construed as a potential conflict of interest.
